# Overexpression of *miR-202* resensitizes imatinib resistant chronic myeloid leukemia cells through targetting Hexokinase 2

**DOI:** 10.1042/BSR20171383

**Published:** 2018-05-08

**Authors:** Yingjun Deng, Xin Li, Jinxin Feng, Xiangliang Zhang

**Affiliations:** 1 Department of Abdominal Surgery, Affiliated Cancer Hospital and Institute of Guangzhou Medical University, Guangzhou, Guangdong, China 510095; 2 Department of Clinical Laboratory, Affiliated Cancer Hospital and Institute of Guangzhou Medical University, Guangzhou, Guangdong,China 510095

**Keywords:** chronic myeloid leukemia, Hexokinase 2, imatinib, miR-202

## Abstract

Chronic myeloid leukemia (CML) is a myeloproliferative disease which uniquely expresses a constitutively active tyrosine kinase, BCR/ABL. As a specific inhibitor of the BCR-ABL tyrosine kinase, imatinib becomes the first choice for the treatment of CML due to its high efficacy and low toxicity. However, the development of imatinib resistance limits the long-term treatment benefits of it in CML patients. In the present study, we aimed to investigate the roles of *miR-202* in the regulation of imatinib sensitivity in CML cell lines and the possible mechanisms involved in this process. We found *miR-202* was down-regulated in seven CML cell lines by quantitative reverse-transcription PCR (qRT-PCR) analysis. Overexpression of *miR-202* significantly suppressed proliferation rates of CML cells. By establishing imatinib resistant cell lines originating from K562 and KU812 cells, we observed expressions of *miR-202* were down-regulated by imatinib treatments and imatinib resistant CML cell lines exhibited lower level of *miR-202*. On the contrary, imatinib resistant CML cell lines displayed up-regulated glycolysis rate than sensitive cells with the evidence that glucose uptake, lactate production, and key glycolysis enzymes were elevated in imatinib resistant cells. Importantly, the imatinib resistant CML cell lines were more sensitive to glucose starvation and glycolysis inhibitors. In addition, we identified Hexokinase 2 (HK2) as a direct target of *miR-202* in CML cell lines. Overexpression of *miR-202* sensitized imatinib resistant CML through the* miR-202*-mediated glycolysis inhibition by targetting HK2. Finally, we provided the clinical relevance that *miR-202* was down-regulated in CML patients and patients with lower *miR-202* expression displayed higher HK2 expression. The present study will provide new aspects on the miRNA-modulated tyrosine kinase inhibitor (TKI) sensitivity in CML, contributing to the development of new therapeutic anticancer drugs.

## Introduction

Chronic myeloid leukemia (CML), originating from a constitutively active tyrosine kinase, BCR/ABL1 which occurs spontaneously, is a myeloproliferative disease affecting older adults typically [1,2]. CML accounts for 15–20% of the newly diagnosed cases of adult leukemias [3]. It is well studied that the BCR/ABL fusion protein is essential and sufficient for the malignant transformation of CML [4,5]. Moreover, the mutant BCR/ABL protein confers malignant differentiation and proliferation of hematopoietic cells, resulting in leukemogenesis [6].

Recent studies have evaluated the molecular and cellular mechanisms contributing to CML. Imatinib is tyrosine kinase inhibitor (TKI), which is first used as a CML treatment strategy through targetting the tyrosine kinase activity of Bcr-Abl [7]. It is a TKI of the 2-phenylamino pyrimidine class through blocking the inactive conformation of BCR-ABL protein [7]. Initially, imatinib treatments displayed high efficacy and low toxicity in CML patients [7]. However, the development of resistance to imatinib treatment which occurs in CML patients greatly impaired the clinical application of imatinib [8,9]. Therefore, investigating the molecular mechanisms of the imatinib resistance is an important task to develop novel therapeutic strategy for improvement of the chemotherapeutic outcomes in CML.

miRNAs are a family non-coding RNAs consisting of 19–24 nts, which affect gene expression through binding to 3′-UTR within target mRNAs [10]. MiRNAs play essential roles in multiple cellular processes such as stem cell division [11], development [12], cellular metabolism [13] and carcinogenesis [14,15]. It has been reported that miRNAs act as either tumor suppressors or oncogenes [14]. Moreover, aberrant miRNAs exhibit a global down-regulation, which is observed in cancers [16], suggesting miRNAs are tumor suppressors at overall level. A growing evidence has revealed specific miRNAs in the pathogenesis of hematological malignancies such as chronic lymphocytic leukemia (CLL), B-cell lymphomas, acute promyelocytic leukemias, acute lymphocytic leukemia (ALL), and CML [17]. Recently, a miRNA microarray study comparing the miRNAs expression in K562 cell line with human healthy blood samples implicated a number of miRNAs were down-regulated in CML cell lines [18], indicating miRNAs might contribute to therapeutics in CML patients.

Previous studies reported that *miR-202* was down-regulated in multiple cancers, such as breast cancer [19], liver cancer [20], gastric cancer [21], pancreatic cancer [22], non-small-cell lung cancer [23], and cervical cancer [24]. Moreover, overexpression of *miR-202* inhibited tumor growth, suggesting that *miR-202* plays suppressive roles in multiple cancer types and might contribute to enhancement of chemotherapy. However, the functions and molecular mechanisms of *miR-202* in human leukemia as well as imatinib sensitivity have not been documented. In the present study, the roles of *miR-202* in mediating imatinib sensitivity will be studied. By comparing the cellular metabolic profiles between K562 imatinib sensitive and resistant cells, the mechanisms of imatinib resistance in CML will be explored. Our study will provide new insights into *miR-202* as a potential molecular target for development of anticancer agents against CML.

## Materials and methods

### Patient samples and ethics

Fifteen patients with newly diagnosed CML (eight males and seven females, aged 19–62 years) were recruited in the present study. None was treated with chemotherapeutic agents before. The control samples were from ten healthy donors (five males and five females, aged 19–60 years). Blood samples from healthy volunteers and CML patients were collected after obtaining informed consents according to procedures approved by the Ethics Committee at Affiliated Cancer Hospital and Institute of Guangzhou Medical University, Guangzhou, China.

### CML cell lines

The human CML cell lines K562 and KU812 were obtained from the American Type Culture Collection (ATCC) (Manassas, VA, U.S.A.). EM2, EM3, LAMA 84, KCL-22, and HL-60 were obtained from the German Resource Center for Biological Material (DSMZ) (Germany). Cells were cultured in RPMI-1640 medium supplemented with 10% heat-inactivated FBS, 2 mM glutamine, 1% penicillin and streptomycin, and cultured at 37°C in a humidified incubator with 5% CO_2_.

### Antibodies and reagents

Antibodies used in the present study were purchased from Cell Signaling Technology (Danvers, MA, U.S.A.): rabbit anti-Glut1 monoclonal antibody: (#12939); rabbit anti-Hexokinase 2 (HK2) monoclonal antibody: (#2867); rabbit anti-LDHA monoclonal antibody: (#3582); total PARP and cleaved PARP: (#9532) mouse anti-β-actin monoclonal antibody: (#3700). Imatinib mesylate, DAPI, 2-deoxyglucose (2-DG), and Oxamate were purchased from Sigma–Aldrich (Shanghai, China).

### Leukocytes isolation

The leukocytes were isolated according to the previous reports [18]. Briefly, peripheral blood samples were drawn from newly diagnosed CML patients and from healthy volunteers. Samples were treated with red blood cell lysis buffer for 30 min. Blood samples were then mixed with erythrocyte lysis buffer (Qiagen, Shanghai, China) and centrifuged at 400 × ***g*** for 10 min at 4°C. The leukocyte pellet was washed and centrifuged again. The remaining leukocytes were collected and frozen for experiments in the present study.

### Real-time PCR for detection of miRNAs and mRNAs

MiRNA real-time RT-PCR was performed using the TaqMan Small RNA primer and probe sets (Applied Biosystems, U.S.A.) according to the manufacturer’s instructions. Total RNA was isolated from cell lines and leukocytes purified from blood of CML patients and of healthy volunteers using TRIzol method according to the previous reports [18]. RNA was reverse-transcribed with miRNA specific stem-looped primers (Applied Biosystems, U.S.A.). Mixture was incubated at 16°C for 30 min; 42°C for 30 min; and 85°C for 5 min. Real-time PCR was performed in duplicates using the following conditions: 95°C for 10 min, followed by 40 cycles of 95°C for 15 s, and 60°C for 1 min. U6-snRNA was used as an internal control. For detection of mRNAs of glycolytic enzymes, the total RNA was isolated from cell lines by TRIzol method. Total RNA (1 μg) of each cell line was reverse transcribed using the High Capacity cDNA Reverse Transcription Kit (Applied Biosystems, Foster City, CA). The cDNA reaction was diluted to 1:10 for use as a template for real-time RT-PCR. The 18S ribosomal primers and probes (Applied Biosystems, Foster City, CA) were used as internal controls. PCR amplifications were performed in a final reaction volume of 20 μl containing, 10 μl of TaqMan Universal PCR Master Mix (Applied Biosystems, Foster City, CA), 1 μl of the primers and probes mix and 9 μl of the cDNA diluted solution. The cycling conditions were as follows: one cycle of 2 min at 50°C, one cycle of 10 min at 95°C, 38 cycles of denaturation (30 s at 95°C) and annealing/extension (1 min at 55°C and 15 s at 72°C). All quantitative PCR reactions were carried out in triplicate and repeated at least twice. Relative mRNA or miRNA expression was calculated using the formula 2^(–ΔΔ*C*^^_t_^^)^. The primers used for real-time PCR were: Glut1 (forward: 5′-AACTCTTCAGCCAGGGTCCAC-3′; reverse: 5′-CACAGTGAAGATGATGAAGAC-3′); HK2 (forward: 5′-CAAAGTGACAGTGGGTGTGG-3′; reverse: 5′-GCCAGGTCCTTCACTGTCTC-3′); and LDHA (forward: 5′-TTGGTCCAGCGTAACGTGAAC-3′; reverse: 5′-CCAGGATGTGTAGCCTTTGAG-3′).

### DAPI staining

Untreated and treated K562 or KU812 imatinib sensitive and resistant cells were harvested by centrifugation and dripped on to a glass slide. Cells were fixed with 4% paraformaldehyde, and membrane permeabilized by exposure for 30 min to 0.1% Triton X-100 in PBS at room temperature. Cells were stained with DNA-binding fluorochrome DAPI for 10 min. The cells were then observed through a fluorescence microscope (Leica Microsystems AG, Wetzlar, Germany).

### miRNA and plasmid DNA transfection

Cells were transfected using the Lipofectamine® LTX Transfection reagent (Invitrogen, Carlsbad, CA) according to the manufacturer’s protocol. miRNA transfection was performed with 100 nM and plasmid DNA was transfected with 2 μg. Forty-eight hours after transfection, cells were collected and prepared for further analysis.

### Glycolysis assay

The glucose uptake and lactate production assays were performed using Glucose Uptake Colorimetric Assay Kit (# K676, Biovision, Milpitas, CA, U.S.A.) and Lactate Colorimetric Assay Kit (#K627, Biovision, Milpitas, CA, U.S.A.) according to the manufacturer’s protocols. The relative glucose uptake/lactate production results were normalized to the amount of total protein compared with the control cells.

### MTT assay

Cells were seeded into a 96-well plate at a density of 5 × 10^3^ cells per well in 200 μl medium and treated under specified glycolysis inhibitors or imatinib for 24 h. After treatments, MTT (5 mg/ml in PBS) was added to each well and incubated for 3-4 h. Suspension cells in the 96-well plate were centrifuged and the MTT solution was removed from the wells by aspiration and the formazan crystals were dissolved in DMSO. Absorbance was measured at 570 nm using a model 450 microplate reader (Bio-Rad Laboratories). Each experiment was performed in triplicate.

### BrdU assay

The BrdU assay was performed using the BrdU Cell Proliferation ELISA Kit (colorimetric) (ab126556, Abcam) according to the manufacturer’s instructions. Absorbance was measured at 450 nm using a model 450 microplate reader (Bio-Rad Laboratories). Each experiment was performed in triplicate.

### Caspase-3 assay

The activity of Caspase-3 was measured using the Caspase 3 Assay Kit (Colorimetric) (ab39401, Abcam) according to the manufacturer’s instructions. Absorbance was measured at 405 nm using a model 450 microplate reader (Bio-Rad Laboratories). Each experiment was performed in triplicate.

### Western blots

Cells were collected and pelleted by centrifugation at 13000×* g* for 10 min at 4°C, washed with ice-cold PBS, and lysed with radioimmunoprecipitation assay (RIPA) buffer (50 mmol/l TrisHCl (pH 7.4), 150 mmol/l NaCl, 2 mmol/l EDTA, 1% NP40, and 0.1% SDS), plus protease inhibitors (complete cocktail tablets; Roche Applied Science). Protein concentration was assessed by the Bradford assay according to the previous description to ensure equal protein loading of each sample. Proteins were separated by SDS/PAGE and transferred to nitrocellulose membrane (Bio-Rad Laboratories) for immunoblotting. Membranes were blocked with 5% BSA solution for 1 h at room temperature. Blots were incubated with primary antibodies at a dilution of 1:1000 at 4°C for overnight. After washing by PBS, membranes were incubated with second antibodies at a dilution of 1:3000 at room temperature for 1 h. Proteins were visualized using the SuperSignal detection substrate (Pierce Biotechnology).

### Statistics analysis

Differences were statistically evaluated by Student’s *t* test using Prism 5.0 software. *P* <0.05 was considered to be statistically significant. Experiments were done in triplicates.

## Results

### *miR-202* is down-regulated in CML cells

**Figure 1:**
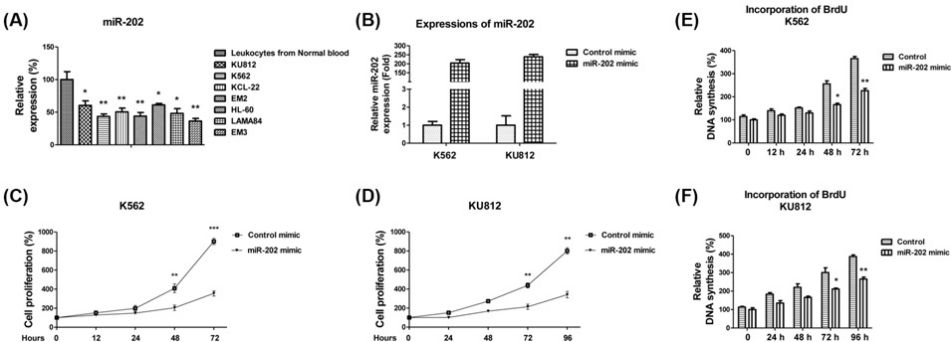
*miR-202* is down-regulated in CML cell lines and suppresses CML cell proliferation (**A**) The expressions of *miR-202* seven CML cell lines and isolated leukocytes from a pool of seven healthy blood samples were measured using qRT-PCR. U6 snRNA was used as a loading control. (**B**) Control mimic or miR-202 mimic was transfected into K562 and KU812 cells for 48 h, followed by the measurements of *miR-202* by qRT-PCR. U6 snRNA was used as a loading control. (**C**) Control mimic or *miR-202* mimic was transfected into K562 and (**D**) KU812 cells for 48 h, followed by the measurements of cell proliferation at 0, 12, 24, 48 and 72 h by MTT assay and (**E**,**F**) BrdU assay. The results are presented as mean ± S.D. of triplicates from each of three independent experiments. *P <0.05; **P <0.01; ***P <0.001.

It has been known that miRNAs play important roles in multiple hematological malignancies [17]. With the objective of deciphering the potential roles of *miR-202* in CML, we analyzed the *miR-202* expression levels in multiple CML cell lines, including KU812, K562, KCL-22, EM2, HL-60, LAMA84, and EM3 and isolated leukocytes from a pool of healthy blood samples. *MiR-202* expressions were then evaluated by quantitative reverse-transcription PCR (qRT-PCR) and our results demonstrated that *miR-202* was significantly down-regulated in seven CML cell lines compared with leukocytes from normal blood sample (Figure 1A), suggesting that *miR-202* might act as a tumor suppressor in CML.

### Overexpression of *miR-202* suppresses proliferation of CML cell lines

The above results showed a strong down-regulation of *miR-202* in CML cell lines. We, therefore, investigated the functional significance of *miR-202* in the regulation of cell proliferation of CML cell lines. We transfected the CML cell line K562 and KU812 with hsa-*miR-202* mimic or control mimic. Compared with the transfection of control mimic, *miR-202* was significantly overexpressed in the K562 and KU812 cells after transfection with *miR-202* mimic (Figure 1B). Compared with control mimic, cells with forced expression of *miR-202* exhibited significantly decreased proliferation from MTT and BrdU assays (Figure 1C–F), supporting that *miR-202* displays tumor suppressive functions in CML cell lines.

### Imatinib resistant cells display down-regulated *miR-202*

**Figure 2:**
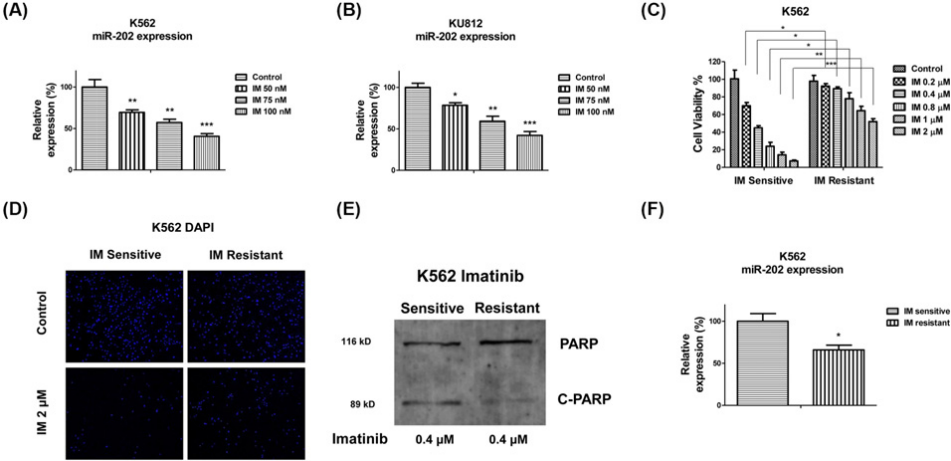
*miR-202* is negatively correlated with imatinib resistance in CML cell lines (**A**) K562 or (**B**) KU812 cells were treated with imatinib at 50, 75 or 100 nM for 24 h, then the expressions of *miR-202* were measured by qRT-PCR. U6 snRNA was used as a loading control. (**C**) K562 imatinib sensitive and resistant cells were treated with imatinib at 0, 0.2, 0.4, 0.8, 1 or 2 μM for 24 h, followed by the measurements of cell viability by MTT assay. (**D**) K562 imatinib sensitive and resistant cells were treated with imatinib at 0 or 2 μM for 24 h, followed by the staining of cell nuclei by DAPI. (**E**) K562 imatinib sensitive and resistant cells were treated with imatinib at 0.4 μM for 24 h, followed by the Western blot analysis. (**F**) The expression of *miR-202* was measured in K562 imatinib sensitive or resistant cells by qRT-PCR. U6 snRNA was used as a loading control. The results are presented as mean ± S.D. of triplicates from each of three independent experiments. *P <0.05; **P <0.01; ***P <0.001.

We continued to explore the functions of *miR-202* in the chemosensitivity of CML cell lines. In the present study, we focused on imatinib which is a commonly used chemotherapeutic agent for CML. Interestingly, in both cell lines, K562-s and KU812, treatment with 1 μM of imatinib for 24 and 48 h significantly suppressed *miR-202* expressions (Figure 2A,B). To further investigate the potential roles of *miR-202* in imatinib sensitivity of CML cell lines, we established imatinib resistant cell line originating from K562 parental CML cell lines according to the previously reported methods [25]. K562 parental cells were treated with gradually increased concentrations of imatinib for three consecutive months. The survival cell clones were saved and pooled for the following experiments in the present study. Results in Figure 2C,D showed the K562 resistant cells could tolerate higher concentrations of imatinib treatments than parental cells. The IC_50_ of K562 resistant cells was 2.16 μM, which is approximately six-fold higher than that of parental cells (0.37 μM). Moreover, the K562 parental cells showed a higher percentage of cleaved PARP than the resistant cells under imatinib treatments (Figure 2E). Taken together, the above results validated the selection of imatinib resistant K562 cell line. In order to observe the direct evidence for supporting the roles of *miR-202* in imatinib resistance, we compared the expressions of *miR-202* in imatinib sensitive and resistant cells. As we expected, *miR-202* is significantly down-regulated in imatinib resistant cells (Figure 2F), indicating *miR-202* might contribute to the sensitization of the CML imatinib.

### Elevated glycolysis in imatinib resistant CML cell lines

**Figure 3:**
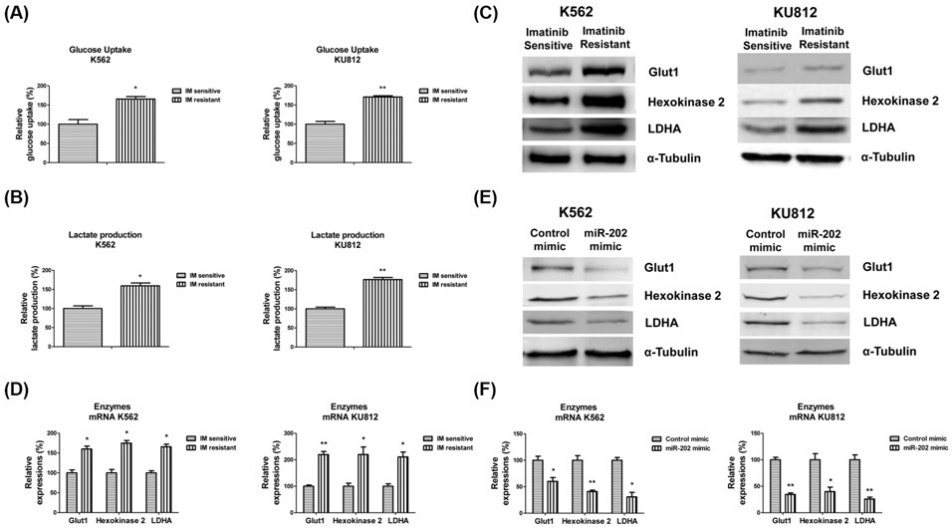
Glycolysis is up-regulated in K562 imatinib resistant cells (**A**) Glucose uptake and (**B**) lactate production were measured in K562 or KU812 imatinib sensitive and resistant cells. (**C**) The protein and (**D**) mRNA expressions of glycolysis enzymes Glut1, HK2, and LDHA were measured in K562 or KU812 imatinib sensitive and resistant cells. (**E**) K562 and KU812 were transfected with control mimic or *miR-202* mimic for 48 h, the protein and (**F**) mRNA expressions of glycolysis enzymes Glut1, HK2, and LDHA were measured. α-Tubulin is a loading control. The results are presented as mean ± S.D. of triplicates from each of three independent experiments. *P <0.05; **P <0.01.

It has been known that the altered glycose metabolism is a biochemical fingerprint of cancer cells and contributes to chemosensitivity [26]. To investigate the mechanisms for the *miR-202*-associated imatinib resistance in CML cell lines, we compared the glycolysis of imatinib sensitive and resistant CML cell lines. The imatinib resistant K562 and KU812 cells showed obviously different glucose metabolism profiles compared with imatinib sensitive cells. The imatinib resistant K562 and KU812 cells exhibited significantly higher levels of glucose uptake and lactate production than sensitive cells (Figure 3A,B). Consistently, we observed the glycolysis key enzymes, Glut1, HK2, and LDHA were up-regulated in imatinib resistant cells at both protein and mRNA levels (Figure 3C,D). Furthermore, to assess whether *miR-202* could regulate glycolysis enzymes expressions, we compared protein and mRNA expression of Glut1, HK2, and LDHA in CML cells with or without *miR-202* overexpression. K562 and KU812 cells with *miR-202* overexpression demonstrated significantly down-regulated glycolysis enzymes expressions (Figure 3E,F). These results demonstrated that the imatinib resistant cells exhibit a highly glycolytic phenotype, suggesting that targetting the dysregulated glycolysis in CML cell lines might contribute to overcome imatinib resistance.

### Imatinib resistant CML cell lines are sensitive to glycolysis inhibitors

**Figure 4:**
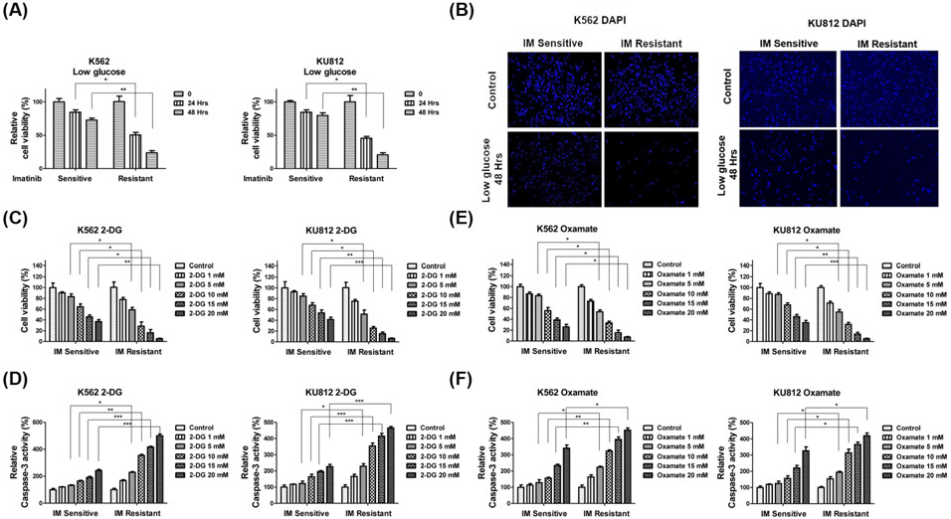
K562 imatinib resistant cells were sensitive to glycolysis inhibition (**A**) K562 or KU812 imatinib sensitive and resistant cells were cultured with low glucose RPMI-1640 medium for 24 or 48 h. The cell viabilities were measured by MTT assay. (**B**) K562 or KU812 imatinib sensitive and resistant cells were cultured with low glucose RPMI-1640 medium for 48 h. Cells were fixed and stained by DAPI. (**C**) K562 or KU812 imatinib sensitive and resistant cells were treated with 2-DG for 24 h, followed by the measurements of cell viability by MTT assay and (**D**) Caspase-3 activity assay. (**E**) K562 or KU812 imatinib sensitive and resistant cells were treated with Oxamate for 48 h, followed by the measurements of cell viability by MTT assay and (**F**) Caspase-3 activity assay. The results are presented as mean ± S.D. of triplicates from each of three independent experiments. *P <0.05; **P <0.01; ***P <0.001.

Since the imatinib resistant CML cell lines have higher glucose metabolic rate, we hypothesized inhibition of glycolysis might sensitize CML cell lines to imatinib. To test it, we first treated both cells with low glucose medium to assess the cell survival rates. As we expected, the imatinib resistant K562 or KU812 cells were more susceptive to low glucose supply. Under low glucose conditions, more than 80% imatinib resistant cells died, lower than that of sensitive cells (25%) (Figure 4A,B). We then treated both imatinib sensitive and resistant K562 or KU812 cells with glycolysis inhibitors, 2-DG or Oxamate. 2-DG is a glucose analog, inhibits glycolysis then glucose cannot be fully oxidized [27] and Oxamate is a cytosolic inhibitor of LDHA [28]. Consistent with the above results in Figure 4A,B, MTT and Caspase-3 activity assays demonstrated imatinib resistant CML cell lines were more sensitive to glycolysis inhibitors compared with imatinib sensitive cells (Figure 4C–F), indicating the combination of glycolysis inhibitor with imatinib might display a synergistically inhibitory effect on CML cell lines.

### *miR-202* inhibits glycolysis through targetting HK2

**Figure 5:**
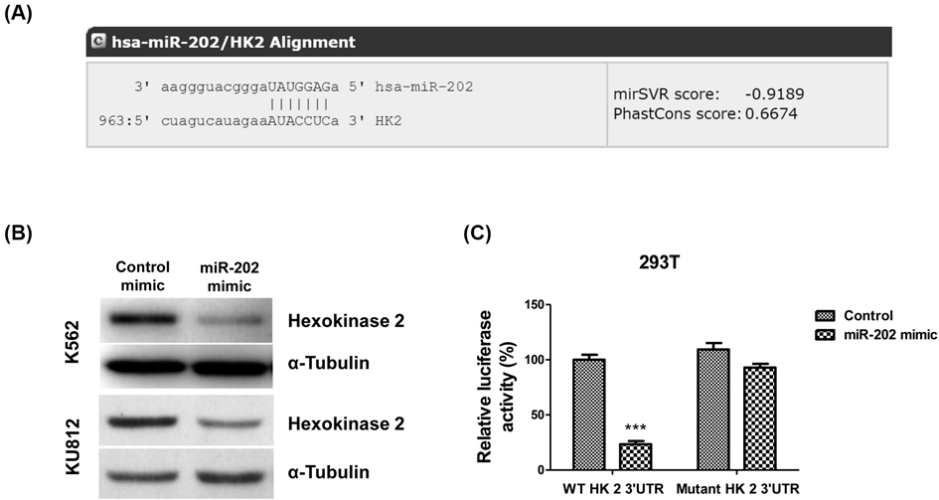
*miR-202* targets HK2 in K562 cells (**A**) The sequence alignment of *miR-202* and 3′-UTR regent of HK2 analyzed from microRNA.org. (**B**) K562 and KU812 cells were transfected with control mimic or *miR-202* mimic for 48 h, followed by Western blot analysis. α-Tubulin is the loading control. (**C**) Luciferase assay showed that *miR-202* could bind 3′-UTR of wild-type HK2, but could not bind to 3′-UTR mutant HK2. The results are presented as mean ± S.D. of triplicates from each of three independent experiments. ***P <0.001.

Our results revealed a negative correlation between *miR-202* and imatinib resistance and a positive correlation between glycolysis and imatinib resistance in CML cell lines. Following that, we investigated whether *miR-202* could directly regulate glycolysis in CML cell lines. By analyzing the 3′-UTR of glycolytic enzymes from public miRNA database  microRNA.org, we observed that the 3′-UTR of HK2 contains *miR-202* binding sites (Figure 5A), suggesting that *miR-202* might directly target HK2. To assess the effect of *miR-202* on HK expression, we performed Western blot analysis. The K562 and KU812 cells were transfected with control mimic or *miR-202* mimic. Results in Figure 5B illustrated that overexpression of *miR-202* inhibited HK2 protein expression. To further explore whether HK2 is a direct target of* miR-202*, we cloned the full-length HK2 3′-UTR or mutant HK2 3′-UTR into a luciferase reporter vector. 293 T cells were co-transfected with control mimic or *miR-202* mimic with vector containing the full-length HK2 3′-UTR or mutant HK2 3′-UTR. As we expected, luciferase assay revealed that *miR-202* directly bound to HK2 3′-UTR, and by which it remarkably reduced luciferase activities (Figure 5C). However, mutation of the putative *miR-202* binding sites in the 3′-UTR of HK2 abrogated luciferase responsiveness to *miR-202* (Figure 5C).

### Overexpression of *miR-202* sensitizes imatinib resistant CML cell lines by inhibiting glycolysis

**Figure 6:**
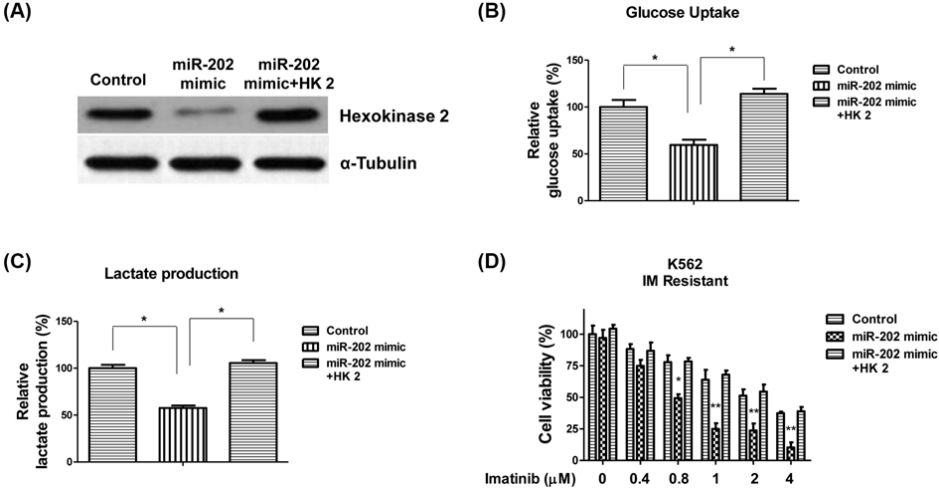
Restoration of HK2 renders K562 cells resistant to imatinib (**A**) K562 cells were transfected with control mimic, *miR-202* mimic, or *miR-202* mimic plus HK2 overexpression vector for 48 h, followed by Western blot analysis. α-Tubulin is the loading control. (**B**) K562 cells were transfected with control mimic, *miR-202* mimic, or *miR-202* mimic plus HK2 overexpression vector for 48 h, followed by measurements of glucose uptake or (**C**) lactate production. (**D**) K562 imatinib resistant cells were transfected with control mimic, *miR-202* mimic, or *miR-202* mimic plus HK2 overexpression vector for 48 h, followed by the treatments of imatinib at 0, 0.4, 0.8, 1, 2 or 4 μM for 24 h. Cell viabilities were measured by MTT assay. The results are presented as mean ± S.D. of triplicates from each of three independent experiments. *P <0.05; **P <0.01.

To understand whether *miR-202* suppresses glycolysis of CML cell lines through directly targetting HK2, we transfected CML cell lines with control mimic, *miR-202* mimic, or *miR-202* mimic with HK2 overexpression vector. Results illustrated co-transfection of *miR-202* mimic with HK2 could restore the original level of HK2 in K562 cells (Figure 6A). Moreover, the glucose uptake and lactate production were recovered by the rescue of HK2 (Figure 6B,C), suggesting *miR-202*-mediated glycolysis inhibition was through targetting HK2. Importantly, our results demonstrated suppression of HK2 by *miR-202* sensitized imatinib resistant K562 cells. Conversely, restoration of HK2 renders *miR-202* overexpressing cells resistant to imatinib (Figure 6D), indicating *miR-202* sensitizes imatinib resistant CML cell lines through targetting HK2.

### Invert correlation of *miR-202* and HK2 expressions in CML patients

**Figure 7:**
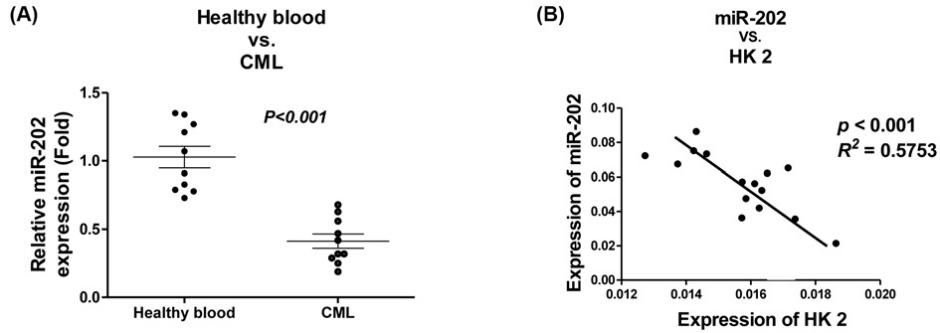
Clinical correlation between *miR-202* and HK2 (**A**) The comparison of *miR-202* expressions in healthy blood samples and CML patient samples. *miR-202* was measured by qRT-PCR. (**B**) The invert expression between *miR-202* and HK2 in CML patient samples. *miR-202* and mRNA of HK2 were measured by qRT-PCR.

We next compared the expressions of *miR-202* in leukocytes from CML patients and normal healthy blood samples. Consistent with *in vitro* results, leukocytes isolated from ten CML blood samples exhibited significantly down-regulation of *miR-202* (Figure 7A). The above *in vitro* results showed *miR-202* could directly target HK2 in CML cell lines (Figure 5). To assess whether these *in vitro* results are also clinically applicable, we analyzed the correlation of *miR-202* and HK2 expressions in 15 CML patient samples by qRT-PCR. We observed a significantly negative correlation between *miR-202* and HK2 expressions (*P* <0.001, *R^2^* = 0.5753) in CML patients (Figure 7B). In summary, this clinical analysis indicated that *miR-202* could target HK2 in CML patients.

## Discussion

In the present study, we report a tumor suppressive role of *miR-202* in CML cell lines and patients. miRNAs and their roles in both normal physiological and disease contexts have been widely studied recently [10–18]. A bulk of evidence indicated that miRNAs play crucial roles in myeloid development and leukemogenesis. For example, *miR-155* and *miR-31* were found to be down-regulated, and some tumor suppressor miRs (*miR-16-1*, *miR-15a*, *miR-101*, *miR-568*) were up-regulated in CML [18]. Another publication reported that *miR-203* was shown to be epigenetically suppressed in CML [29], suggesting a tumor inhibitory function of *miR-203* in CML.* MiR-202* has been reported as a tumor suppressor miRNA and was obviously down-regulated in breast carcinoma [19], gastric cancer [21], and lung cancer [23]. Moreover, aberrant expression of *miR-202* is associated with tumorigenesis, tumor progression, and metastasis in multiple cancer types [19–24], suggesting overexpressing *miR-202* contributes to development of novel anticancer agent. Up to date, however, little study described the role of *miR-202* in CML as well as imatinib chemosensitivity. In the present study, we observed that *miR-202* was significantly down-regulated in CML cell lines and patients compared with normal blood cells. Moreover, overexpression of *miR-202* could suppress proliferation, migration and increase chemosensitivity of CML cell lines. Taken together, our study reveals a novel role of *miR-202* in CML.

In 1926, Otto Warburg proposed that cancer cells exhibit high glycolysis even in the presence of oxygen, which was one of the hallmark of cancer development [30]. Metabolic changes in cancer have been recognized with crucial roles in tumorigenesis. Cancer cells up-regulate the rate-limiting enzymes of glycolysis such as GLUT1, HK2, PKM2, and LDHA as a result of the expression of oncogenes including RAS, SRC, or EGFR [31]. In addition, it is widely studied that the dysregulated glucose metabolism contributes to chemoresistance [31]. Consistently, our data demonstrated imatinib resistant CML cell lines displayed up-regulated glycolytic rates and were sensitive to glycolysis inhibitors, suggesting inhibition of glycolysis in CML chemoresistant cells might be a novel therapeutic strategy.

Imatinib is a commonly used chemotherapeutic agent that targets the tyrosine kinase activity of Bcr-Abl because of its high efficacy, low toxicity, and ability to maintain durable hematological responses [7]. However, the development of resistance to imatinib became problems over time. Currently, several mechanisms for the imatinib resistance observed in patients with CML have been studied such as mutations on the BCR-ABL kinase domain, increased BCR-ABL expression, and overexpression of drug-efflux proteins (ABCB1 and ABCG2) [32]. A recent publication reported that glycolysis was highly correlated with imatinib resistance in CML [33], consistent with our observations in this study. In addition, we demonstrated *miR-202* could directly target HK2, which is an essential glycolysis enzyme in the glucose metabolism pathway. We further reported overexpression of *miR-202* sensitized imatinib resistant CML cell lines, suggesting *miR-202* might be a novel therapeutic target against imatinib resistance. Although the detailed mechanisms for the imatinib regulated *miR-202* are still not fully understood, our continuing project will use *in vivo* model to further illustrate the axis of *miR-202*-HK2-glycolysis-imatinib sensitivity in CML.

## Abbreviations

CML: chronic myeloid leukemia
HK2: hexokinase 2
qRT-PCR: quantitative reverse-transcription PCR
TKI: tyrosine kinase inhibitor
2-DG: 2-deoxyglucose
